# 30-day morbidity and mortality of sleeve gastrectomy, Roux-en-Y gastric bypass and one anastomosis gastric bypass: a propensity score-matched analysis of the GENEVA data

**DOI:** 10.1038/s41366-021-01048-1

**Published:** 2021-12-15

**Authors:** Rishi Singhal, Victor Roth Cardoso, Tom Wiggins, Jonathan Super, Christian Ludwig, Georgios V. Gkoutos, Kamal Mahawar, GENEVA Collaborators, Michał Pędziwiatr, Piotr Major, Piotr Zarzycki, Athanasios Pantelis, Dimitris P. Lapatsanis, Georgios Stravodimos, Chris Matthys, Marc Focquet, Wouter Vleeschouwers, Antonio G. Spaventa, Carlos Zerrweck, Antonio Vitiello, Giovanna Berardi, Mario Musella, Alberto Sanchez-Meza, Felipe J. Cantu, Fernando Mora, Marco A. Cantu, Abhishek Katakwar, D. Nageshwar Reddy, Haitham Elmaleh, Mohammad Hassan, Abdelrahman Elghandour, Mohey Elbanna, Ahmed Osman, Athar Khan, Laurent layani, Nalini Kiran, Andrey Velikorechin, Maria Solovyeva, Hamid Melali, Shahab Shahabi, Ashish Agrawal, Apoorv Shrivastava, Ankur Sharma, Bhavya Narwaria, Mahendra Narwaria, Asnat Raziel, Nasser Sakran, Sergio Susmallian, Levent Karagöz, Murat Akbaba, Salih Zeki Pişkin, Ahmet Ziya Balta, Zafer Senol, Emilio Manno, Michele Giuseppe Iovino, Ahmed Osman, Mohamed Qassem, Sebastián Arana-Garza, Heitor P. Povoas, Marcos Leão Vilas-Boas, David Naumann, Alan Li, Basil J. Ammori, Hany Balamoun, Mohammed Salman, Amrit Manik Nasta, Ramen Goel, Hugo Sánchez-Aguilar, Miguel F. Herrera, Adel Abou-mrad, Lucie Cloix, Guilherme Silva Mazzini, Leonardo Kristem, Andre Lazaro, Jose Campos, Joaquín Bernardo, Jesús González, Carlos Trindade, Octávio Viveiros, Rui Ribeiro, David Goitein, David Hazzan, Lior Segev, Tamar Beck, Hernán Reyes, Jerónimo Monterrubio, Paulina García, Marine Benois, Radwan Kassir, Alessandro Contine, Moustafa Elshafei, Sueleyman Aktas, Sylvia Weiner, Till Heidsieck, Luis Level, Silvia Pinango, Patricia Martinez Ortega, Rafael Moncada, Victor Valenti, Ivan Vlahović, Zdenko Boras, Arnaud Liagre, Francesco Martini, Gildas Juglard, Manish Motwani, Sukhvinder Singh Saggu, Hazem Al Momani, Luis Adolfo Aceves López, María Angelina Contreras Cortez, Rodrigo Aceves Zavala, Christine D’Haese RN, Ivo Kempeneers, Jacques Himpens, Andrea Lazzati, Luca Paolino, Sarah Bathaei, Abdulkadir Bedirli, Aydın Yavuz, Çağr Büyükkasap, Safa Özaydın, Andrzej Kwiatkowski, Katarzyna Bartosiak, Maciej Walędziak, Antonella Santonicola, Luigi Angrisani, Paola Iovino, Rossella Palma, Angelo Iossa, Cristian Eugeniu Boru, Francesco De Angelis, Gianfranco Silecchia, Abdulzahra Hussain, Srivinasan Balchandra, Izaskun Balciscueta Coltell, Javier Lorenzo Pérez, Ashok Bohra, Altaf K. Awan, Brijesh Madhok, Paul C. Leeder, Sherif Awad, Waleed Al-Khyatt, Ashraf Shoma, Hosam Elghadban, Sameh Ghareeb, Bryan Mathews, Marina Kurian, Andreas Larentzakis, Gavriella Zoi Vrakopoulou, Konstantinos Albanopoulos, Ahemt Bozdag, Azmi Lale, Cuneyt Kirkil, Mursid Dincer, Ahmad Bashir, Ashraf Haddad, Leen Abu Hijleh, Bruno Zilberstein, Danilo Dallago de Marchi, Willy Petrini Souza, Carl Magnus Brodén, Hjörtur Gislason, Kamran Shah, Antonio Ambrosi, Giovanna Pavone, Nicola Tartaglia, S. Lakshmi Kumari Kona, K. Kalyan, Cesar Ernesto Guevara Perez, Miguel Alberto Forero Botero, Adrian Covic, Daniel Timofte, Madalina Maxim, Dashti Faraj, Larissa Tseng, Ronald Liem, Gürdal Ören, Evren Dilektasli, Ilker Yalcin, Hudhaifa AlMukhtar, Mohammed Al Hadad, Rasmi Mohan, Naresh Arora, Digvijaysingh Bedi, Claire Rives-Lange, Jean-Marc Chevallier, Tigran Poghosyan, Hugues Sebbag, Lamia Zinaï, Saadi Khaldi, Charles Mauchien, Davide Mazza, Georgiana Dinescu, Bernardo Rea, Fernando Pérez-Galaz, Luis Zavala, Anais Besa, Anna Curell, Jose M. Balibrea, Carlos Vaz, Luis Galindo, Nelson Silva, José Luis Estrada Caballero, Sergio Ortiz Sebastian, João Caetano Dallegrave Marchesini, Ricardo Arcanjo da Fonseca Pereira, Wagner Herbert Sobottka, Felipe Eduardo Fiolo, Matias Turchi, Antonio Claudio Jamel Coelho, Andre Luis Zacaron, André Barbosa, Reynaldo Quinino, Gabriel Menaldi, Nicolás Paleari, Pedro Martinez-Duartez, Gabriel Martínez de Aragon Ramírez de Esparza, Valentin Sierra Esteban, Antonio Torres, Jose Luis Garcia-Galocha, Miguel Josa, Jose Manuel Pacheco-Garcia, Maria Angeles Mayo-Ossorio, Pradeep Chowbey, Vandana Soni, Hercio Azevedo de Vasconcelos Cunha, Michel Victor Castilho, Rafael Meneguzzi Alves Ferreira, Thiago Alvim Barreiro, Alexandros Charalabopoulos, Elias Sdralis, Spyridon Davakis, Benoit Bomans, Giovanni Dapri, Koenraad Van Belle, Mazen Takieddine, Pol Vaneukem, Esma Seda Akalın Karaca, Fatih Can Karaca, Aziz Sumer, Caghan Peksen, Osman Anil Savas, Elias Chousleb, Fahad Elmokayed, Islam Fakhereldin, Hany Mohamed Aboshanab, Talal Swelium, Ahmad Gudal, Lamees Gamloo, Ayushka Ugale, Surendra Ugale, Clara Boeker, Christian Reetz, Ibrahim Ali Hakami, Julian Mall, Andreas Alexandrou, Efstratia Baili, Zsolt Bodnar, Almantas Maleckas, Rita Gudaityte, Cem Emir Guldogan, Emre Gundogdu, Mehmet Mahir Ozmen, Deepti Thakkar, Nandakishore Dukkipati, Poonam Shashank Shah, Shashank Subhashchandra Shah, Simran Shashank Shah, Md Tanveer Adil, Periyathambi Jambulingam, Ravikrishna Mamidanna, Douglas Whitelaw, Md Tanveer Adil, Vigyan Jain, Deepa Kizhakke Veetil, Randeep Wadhawan, Antonio Torres, Max Torres, Tabata Tinoco, Wouter Leclercq, Marleen Romeijn, Kelly van de Pas, Ali K. Alkhazraji, Safwan A. Taha, Murat Ustun, Taner Yigit, Aatif Inam, Muhammad Burhanulhaq, Abdolreza Pazouki, Foolad Eghbali, Mohammad Kermansaravi, Amir Hosein Davarpanah Jazi, Mohsen Mahmoudieh, Neda Mogharehabed, Gregory Tsiotos, Konstantinos Stamou, Francisco J. Barrera Rodriguez, Marco A. Rojas Navarro, Omar Mohamed Torres, Sergio Lopez Martinez, Elda Rocio Maltos Tamez, Gustavo A. Millan Cornejo, Jose Eduardo Garcia Flores, Diya Aldeen Mohammed, Mohamad Hayssam Elfawal, Asim Shabbir, Kim Guowei, Jimmy By So, Elif Tuğçe Kaplan, Mehmet Kaplan, Tuğba Kaplan, DangTuan Pham, Gurteshwar Rana, Mojdeh Kappus, Riddish Gadani, Manish Kahitan, Koshish Pokharel, Alan Osborne, Dimitri Pournaras, James Hewes, Errichetta Napolitano, Sonja Chiappetta, Vincenzo Bottino, Evelyn Dorado, Axel Schoettler, Daniel Gaertner, Katharina Fedtke, Francisco Aguilar-Espinosa, Saul Aceves-Lozano, Alessandro Balani, Carlo Nagliati, Damiano Pennisi, Andrea Rizzi, Francesco Frattini, Diego Foschi, Laura Benuzzi, Chirag Parikh, Harshil Shah, Enrico Pinotti, Mauro Montuori, Vincenzo Borrelli, Jerome Dargent, Catalin A. Copaescu, Ionut Hutopila, Bogdan Smeu, Bart Witteman, Eric Hazebroek, Laura Deden, Laura Heusschen, Sietske Okkema, Theo Aufenacker, Willem den Hengst, Wouter Vening, Yonta van der Burgh, Ahmad Ghazal, Hamza Ibrahim, Mourad Niazi, Bilal Alkhaffaf, Mohammad Altarawni, Giovanni Carlo Cesana, Marco Anselmino, Matteo Uccelli, Stefano Olmi, Christine Stier, Tahsin Akmanlar, Thomas Sonnenberg, Uwe Schieferbein, Alejandro Marcolini, Diego Awruch, Marco Vicentin, Eduardo Lemos de Souza Bastos, Samuel Azenha Gregorio, Anmol Ahuja, Tarun Mittal, Roel Bolckmans, Tom Wiggins, Clément Baratte, Judith Aron Wisnewsky, Laurent Genser, Lynn Chong, Lillian Taylor, Salena Ward, Michael W. Hi, Helen Heneghan, Naomi Fearon, Andreas Plamper, Karl Rheinwalt, Helen Heneghan, Justin Geoghegan, Kin Cheung Ng, Naomi Fearon, Krzysztof Kaseja, Maciej Kotowski, Tarig A. Samarkandy, Adolfo Leyva-Alvizo, Lourdes Corzo-Culebro, Cunchuan Wang, Wah Yang, Zhiyong Dong, Manel Riera, Rajesh Jain, Hosam Hamed, Mohammed Said, Katia Zarzar, Manuel Garcia, Ahmet Gökhan Türkçapar, Ozan Şen, Edoardo Baldini, Luigi Conti, Cacio Wietzycoski, Eduardo Lopes, Tadeja Pintar, Jure Salobir, Cengiz Aydin, Semra Demirli Atici, Anıl Ergin, Huseyin Ciyiltepe, Mehmet Abdussamet Bozkurt, Mehmet Celal Kizilkaya, Nezihe Berrin Dodur Onalan, Mariana Nabila Binti Ahmad Zuber, Wei Jin Wong, Amador Garcia, Laura Vidal, Marc Beisani, Jorge Pasquier, Ramon Vilallonga, Sharad Sharma, Chetan Parmar, Lyndcie Lee, Pratik Sufi, Hüseyin Sinan, Mehmet Saydam

**Affiliations:** 1 Upper GI Unit, University Hospital Birmingham NHS Foundation Trust, Birmingham, UK; 2 Institute of Cancer and Genomic Sciences, University of Birmingham, Birmingham, UK; 3 Health Data Research UK Midlands, Birmingham, UK; 4 General Surgery Department, Maidstone and Tunbridge Wells NHS Trust, Tunbridge Wells, UK; 5 Institute of Metabolism and Systems Research, College of Medical and Dental Sciences, University of Birmingham, Birmingham, UK; 6 NIHR Experimental Cancer Medicine Centre, Birmingham, B15 2TT UK; 7 NIHR Surgical Reconstruction and Microbiology Research Centre, Birmingham, B15 2TT UK; 8 Bariatric Unit, South Tyneside and Sunderland NHS Trust, Sunderland, UK; 9 2nd Department of General Surgery, Jagiellonian University Medical College, Krakow, Poland; 10 4th Surgical Department, Evaggelismos General Hospital of Athens, Athens, Greece; 11 AZ Sint Elisabeth Zottegem, Zottegem, Belgium; 12 ABC Medical Center Santa Fe, Mexico City, Mexico; 13 Advanced Biomedical Sciences Department, Naples “Federico II” University, Naples, Italy; 14 Advanced Medicine Institute, Reynosa, Mexico; 15 AIG Hospital, Hyderabad, India; 16 Ain Shams University Hospitals, Cairo, Egypt; 17 Al Shark Hospital, Fujairah, United Arab Emirates; 18 American Medical Clinic, Saint Petersburg, Russia; 19 Amin University Hospital, Isfahan, Iran; 20 Apoorv Hi Tech at Gokuldas Hospital, Indore, India; 21 Asian Bariatrics, Ahmedabad, India; 22 Assuta Medical Center, Tel Aviv, Israel; 23 Atasam Hospitals, Samsun, Turkey; 24 AZBariatrics Obesity Center, Istanbul, Turkey; 25 Bariatric and Metabolic Surgery Unit, Ospedale A. Cardarelli, Naples, Italy; 26 Bariatric Surgery Department, Faculty of Medicine, Ain Shams University, Cairo, Egypt; 27 Bariatric Surgery Experts, Monterrey, Mexico; 28 BAROS—Bariatric and Metabolic Surgery, Salvador, Brazil; 29 Birmingham Heartlands Hospital, University Hospital Birmingham NHS Foundation Trust, Birmingham, UK; 30 BMI Alexandra Hospital, Manchester, UK; 31 Burjeel Hospital, Abu Dhabi, United Arab Emirates; 32 Cairo University, Cairo, Egypt; 33 Center of Metabolic Surgery, Wockhardt Hospital, Agripada, Mumbai, India; 34 Center of Nutrition and Obesity, ABC Medical Center (Observatorio), Mexico City, Mexico; 35 Centre Hospitalier Regional d’ORLEANS, Orléans, France; 36 Centro de Obesidade do Instituto do Aparelho Digestivo, Porto Alegre, Brazil; 37 Centro Hospitalar e Universitario de Coimbra, Coimbra, Portugal; 38 Centro Médico de Asturias, Oviedo, Spain; 39 Centro Multidisciplinar da Doença Metabólica, Clínica Santo Antonio, Lusiadas, Amadora, Portugal; 40 Chaim Sheba Medical Center, Affiliated with Sackler School of Medicine, Tel Aviv University, Ramat Gan, Israel; 41 Christus Muguerza Sur, Monterrey, Mexico; 42 CHU Félix Guyon, la Réunion, Réunion, France; 43 Città di Castello Hospital, Usl Umbria 1, Città di Castello, Italy; 44 Clinic for Metabolic Surgery, Krankenhaus Nordwest, Frankfurt, Germany; 45 Clínica Santa Sofía, Caracas, Venezuela; 46 Clinica Universidad de Navarra, Pamplona, Spain; 47 Clinical Hospital Centre Osijek, Osijek, Croatia; 48 Clinique des Cedres, Cornebarrieu, France; 49 COMS, Apollo Spectra Hospital, New Delhi, India; 50 Danat Al Emarat Hospital, Abu Dhabi, United Arab Emirates; 51 Defeat Obesity Bariatric and Metabolic Surgery, CHRISTUS MUGUERZA Hospital Reynosa, Reynosa, TAMPS Mexico; 52 Delta CHIREC Hospital, Brussels, Belgium; 53 Department of General Surgery, Center Hospitalier Intercommunal de Créteil, Paris, France; 54 Department of General Surgery, Gazi University Faculty of Medicine, Yenimahalle/Ankara, Turkey; 55 Department of General Surgery, Military Institute of Medicine, Szaserów 128, 04-141 Warsaw, Poland; 56 Department of Public Health, “Federico II” University of Naples, Naples, Italy; 57 Division of General Surgery and Bariatric Center of Excellence IFSO-EC, University La Sapienza of Rome, Rome, Italy; 58 Doncaster and Bassetlaw Teaching Hospitals, Yorkshire, UK; 59 Dr. Lorenzo, Innovación Cirugía Obesidad y Diabetes, Valencia, Spain; 60 East-Midlands Bariatric and Metabolic Institute (EMBMI), Royal Derby Hospital, Derby, UK; 61 Elsafa Private Hospital and Mansoura University Hospital and Eldelta Hospital, Mansoura, Egypt; 62 New York Minimally Invasive Surgery PLLC, New York, NY USA; 63 First Department of Propaedeutic Surgery, Hippocration General Athens Hospital, National and Kapodistrian University of Athens, Athens, Greece; 64 Fırat University Hospital, Elazığ, Turkey; 65 Gastrointestinal, Bariatric and Metabolic Center at Jordan Hospital, Amman, Jordan; 66 GASTROMED-Zilberstein Institute, Sao Paulo, Brazil; 67 GB Obesitas Skaane, Malmö, Sweden; 68 General Surgery, University of Foggia, Foggia, Italy; 69 Glenagles Global Hospital, Lakdikapul, Hyderabad, India; 70 Grammo SAS IPS, Bogotá, Colombia; 71 Grigore T. Popa University of Medicine and Pharmacy, Iasi, Romania; 72 Groene Hart Hospital in Gouda and Dutch Obesity Clinic, The Hague, The Netherlands; 73 Gürdal Ören Bariatric Surgery Center, İstanbul, Turkey; 74 Hayat Hospital, General Surgery, Bariatric and Metabolic Surgery, Bursa, Turkey; 75 Healthpoint Hospital, Abu Dhabi, United Arab Emirates; 76 Hope Obesity Centre, Ahmedabad, India; 77 Hôpital Européen Georges Pompidou, AP-HP, Université de Paris, Paris, France; 78 Hôpital Privé de Provence (HPP), Aix-en-Provence, France; 79 Hôpital Ste Musse Centre Hospitalier, Toulon, France; 80 Hospital Ángeles Lomas, Estado de México, México; 81 Hospital Christus Muguerza Sur, Monterrey, México; 82 Hospital Clínic de Barcelona, Barcelona, Spain; 83 Hospital CUF Tejo, Lisbon, Portugal; 84 Hospital General Universitario Alicante Spain, Alicante, Spain; 85 Hospital Marcelino Champagnat, Curitiba, Brazil; 86 Hospital Privado de Comunidad, Mar del Plata, Argentina; 87 Hospital Rios D’Or, Rio de Janeiro, Brazil; 88 Hospital Unimed Natal, Natal, Brazil; 89 Hospital Universitario Austral, Bariatric and Metabolic Department, Buenos Aires, Argentina; 90 Hospital Universitario de Álava, Vitoria-Gasteiz, Spain; 91 Hospital Universitario Madrid Monteprincipe, Hospital Clinico San Carlos, Madrid, Spain; 92 Hospital Universitario Puerta del Mar, Cadiz, Spain; 93 Institute of Minimal Access, Metabolic and Bariatric Surgery, Max Super-Speciality Hospital, Saket, New Delhi, India; 94 Instituto Campineiro de Tratamento da Obesidade, Campinas, Brazil; 95 Interbalcan Medical Center, Pilea, Greece; 96 International School Reduced Scar Laparoscopy, Brussels, Belgium; 97 Isppc chu-André Vésale, Metabolic and Bariatric Surgery, Montigny-le-Tilleul, Belgium; 98 İstanbul Bilgi University,Turkey (first author), Department of Pulmonary Medicine, Istanbul Yedikule Chest Diseases and Thoracic Surgery Education and Research Hospital (second author), Zeytinburnu, Turkey; 99 Istinye University, School of Medicine, Istanbul, Turkey; 100 Jackson North Medical Center, Miami, Fl USA; 101 King Abdul Aziz Hospital, Alhasa, Saudi Arabia; 102 King Abdullah Medical Complex, Jeddah, Saudi Arabia; 103 Kirloskar Hospital, Hyderabad, India; 104 Klinikum Region Hannover-Klinikum Nordstadt, Hannover, Germany; 105 Laiko General Hospital, National and Kapodistrian University of Athens, Athens, Greece; 106 Letterkenny University Hospital, Letterkenny, Ireland; 107 Lithuanian University of Health Sciences, Surgery Department, Kaunas, Lithuania; 108 Liv Hospital Ankara, Ankara, Turkey; 109 Livlife Hospitals, Hyderabad, India; 110 LOC Healthcare LLP, Pune, India; 111 Luton and Dunstable Hospital, Luton, UK; 112 Luton and Dunstable University Hospital, Luton, UK; 113 Manipal Hospital, New Delhi, India; 114 Max Medical, Centro de Cirugía Bariátrica/Robótica, Hospital Metropilitano de Quito/Ecuador, Quito, Ecuador; 115 Máxima Medical Center, Veldhoven, The Netherlands; 116 Mediclinic Hospital Airport Road, Abu Dhabi, United Arab Emirates; 117 Memorial Hospital, Istanbul, Turkey; 118 Metabolic, Thoracic and General Surgery Team III, Department of General Surgery, Pakistan Institute of Medical Sciences (PIMS), Islamabad, Pakistan; 119 Minimally Invasive Surgery Research Center, Division of Minimally Invasive and Bariatric Surgery, Department of Surgery, Rasool-e Akram Hospital, Iran University of Medical Sciences, Tehran, Iran; 120 Minimally Invasive Surgery Research Center, Isfahan University of Medical Sciences, Isfahan, Iran; 121 MITERA Hospital, Athens, Greece; 122 Monterrey Gastro and Bariatric Group, Monterrey, Mexico; 123 MtyBariatrics, Monterrey, NL Mexico; 124 Najjar Hospital, Beirut, Lebanon; 125 National University Hospital Singapore, Singapore, Singapore; 126 NCR International Hospital, Gaziantep, Turkey; 127 Niagara Falls Memorial Medical Center, Niagara Falls, NY USA; 128 Nobesity Bariatric Centre, KD Hospital, Ahmedabad, India; 129 North Bristol NHS Trust, Bristol, UK; 130 Obesity and Metabolic Surgery Unit, Ospedale Evangelico Betania, Naples, Italy; 131 Obesity and Aesthetic Surgery Clinic Clinica MED, Cali, Colombia; 132 Obesity Center, Municipal Hospital Karlsruhe, Karlsruhe, Germany; 133 Obesity Clinic: Los Altos Obesity Surgery, Tepatitlan, Mexico; 134 Ospedale di Gorizia, Italy, Struttura Complessa Chirurgia Generale, Gorizia, Italy; 135 Ospedale Galmarini Tradate, Varese, Italy; 136 Ospedale San Giuseppe IRCCS Multimedica, University of Milan, Milan, Italy; 137 Parul Institute of Medical Sciences and Research, Parul University, Waghodia, Vadodara, India; 138 Policlinico San Pietro, Unitá di Chirurgia Bariatrica, Bergamo, Italy; 139 Polyclinique Lyon-Nord, 69140 Rillieux, France; 140 Ponderas Academic Hospital, Bucharest, Romania; 141 Rijnstate Hospital/Vitalys Clinics, Arnhem, The Netherlands; 142 Saint Louis Hospital, Aleppo, Aleppo, Syria; 143 Salford Royal NHS Foundation Trust, Salford, UK; 144 San Marco Hospital GSD, Zingonia, BG Italy; 145 Sana Obesity Center Northrhine Westphalia, Clinic for General, Visceral, and Transplantation Surgery, RWTH University Aachen, Aachen, Germany; 146 Sana Obesity Center Northrhine Westphalia, Westphalia, Germany; 147 Sanatorio Britanico de Rosario, Rosario, Santa Fe, Argentina; 148 Santa Casa de Marilia, Marilia, Brazil; 149 Sir Ganga Ram Hospital, Delhi, India; 150 Somerset NHS Foundation Trust, Taunton, UK; 151 Sorbonne Université, Institute of Cardiometabolism and Nutrition ICAN, Assistance Publique-Hôpitaux de Paris, Departments of Digestive surgery and Nutrition, Pitié-Salpêtrière University Hospital, Paris, France; 152 St. Vincent’s Hospital Melbourne, Fitzroy, Australia; 153 St. Vincent’s University Hospital, Dublin, Ireland; 154 St. Franziskus Hospital, Cologne, Germany; 155 State Clinical Hospital No. 2 of the Pomeranian Medical University in Szczecin, Szczecin, Poland; 156 Sutter Gould Medical Foundation, Dameron Hospital, Stockton, CA USA; 157 Tecnologico de Monterrey, Monterrey, MX Mexico; 158 The First Affiliated Hospital of Jinan University, Guangzhou, China; 159 The Shrewsbury and Telford Hospital, Shrewsbury, UK; 160 Truelife Bariatric and Digestive Surgery Center, Mansoura, Dakahleyya, Egypt; 161 Tu Opcion Bariatrica, Monterrey, Mexico; 162 Türkçapar Bariatrics Obesity Center, İstanbul, Turkey; 163 U.O. Chirurgia, Ospedale “Guglielmo da Saliceto”, Piacenza, Italy; 164 Unimed Vale do Caí Hospital, Montenegro, BR. Maicé Hospital, Caçador, BR Brazil; 165 University Medical Center Ljubljana, Ljubljana, Slovenia; 166 University of Health Sciences Tepecik Training and Research Hospital, Department of General Surgery, Izmir, Turkey; 167 University of Health Sciences, Fatih Sultan Mehmet Training and Research Hospital, General Surgery Department, Istanbul, Turkey; 168 University of Health Sciences, Kanuni Sultan Süleyman Training and Research Hospital, Istanbul, Turkey; 169 University of Malaya Medical Centre, Kuala Lumpur, Malaysia; 170 Vall d’Hebron University Hospital, Barcelona, Spain; 171 Vall Hebron Hospital Campus—Hospital de Barcelonoa-SCIAS, Barcelona, Spain; 172 Vinamra Swaraj Hospital, Navi Mumbai, India; 173 Whittington Health NHS Trust, London, UK; 174 Department of Metabolic Surgery, Special Etiler Hospital, Istanbul, Turkey; 175 Department of General Surgery, Diskapi Yildirim Beyazit Training and Research Hospital, Ankara, Turkey

**Keywords:** Obesity, Obesity

## Abstract

**Background:**

There is a paucity of data comparing 30-day morbidity and mortality of sleeve gastrectomy (SG), Roux-en-Y gastric bypass (RYGB), and one anastomosis gastric bypass (OAGB). This study aimed to compare the 30-day safety of SG, RYGB, and OAGB in propensity score-matched cohorts.

**Materials and methods:**

This analysis utilised data collected from the GENEVA study which was a multicentre observational cohort study of bariatric and metabolic surgery (BMS) in 185 centres across 42 countries between 01/05/2022 and 31/10/2020 during the Coronavirus Disease-2019 (COVID-19) pandemic. 30-day complications were categorised according to the Clavien–Dindo classification. Patients receiving SG, RYGB, or OAGB were propensity-matched according to baseline characteristics and 30-day complications were compared between groups.

**Results:**

In total, 6770 patients (SG 3983; OAGB 702; RYGB 2085) were included in this analysis. Prior to matching, RYGB was associated with highest 30-day complication rate (SG 5.8%; OAGB 7.5%; RYGB 8.0% (*p* = 0.006)). On multivariate regression modelling, Insulin-dependent type 2 diabetes mellitus and hypercholesterolaemia were associated with increased 30-day complications. Being a non-smoker was associated with reduced complication rates. When compared to SG as a reference category, RYGB, but not OAGB, was associated with an increased rate of 30-day complications. A total of 702 pairs of SG and OAGB were propensity score-matched. The complication rate in the SG group was 7.3% (*n* = 51) as compared to 7.5% (*n* = 53) in the OAGB group (*p* = 0.68). Similarly, 2085 pairs of SG and RYGB were propensity score-matched. The complication rate in the SG group was 6.1% (*n* = 127) as compared to 7.9% (*n* = 166) in the RYGB group (*p* = 0.09). And, 702 pairs of OAGB and RYGB were matched. The complication rate in both groups was the same at 7.5 % (*n* = 53; *p* = 0.07).

**Conclusions:**

This global study found no significant difference in the 30-day morbidity and mortality of SG, RYGB, and OAGB in propensity score-matched cohorts.

## Introduction

Sleeve gastrectomy (SG), Roux-en-Y gastric bypass (RYGB), and one anastomosis gastric bypass (OAGB) are the three commonest bariatric procedures worldwide [1]. There is currently no randomised controlled trial (RCT) comparing these three procedures in the scientific literature. There are several RCTs comparing two of these three procedures [2, 3] but they were not powered to evaluate differences in morbidity or mortality.

30-day morbidity and mortality is a recognised outcome measure for the evaluation of surgical safety and has been used in surgical literature for several decades [4]. There are large studies comparing 30-day morbidity and mortality of RYGB and SG. Alizadeh et al. [5] reported from an analysis of Metabolic and Bariatric Surgery Accreditation and Quality Improvement Program (MBSAQIP) data in the United States that RYGB was associated with higher 30-day morbidity (4.4% vs 2.3%; adjusted odds ratio (AOR) 0.53; *p* < 0.01) and 30-day mortality (0.2% vs 0.1%; AOR 0.58; *p* = 0.07) in comparison with SG. However, there is no large data in the scientific literature comparing 30-day morbidity and mortality of SG and OAGB or RYGB and OAGB.

Notwithstanding the lack of such large data, these direct database comparisons are often flawed due to significant differences in the baseline population. Propensity score matching is a valid tool for comparing non-randomised populations by matching them for confounding variables [6]. To the best of our knowledge, there is only one published study comparing 30-day morbidity and mortality of SG and RYGB [7]; and one comparing RYGB and OAGB [8] in propensity score-matched populations. Both of these studies emanate from the MBSAQIP database. In their study, Kapur et al. [7] found lower adverse events with SG in comparison with RYGB. However, the study by Docimo et al. [8] comparing the 30-day morbidity of OAGB and RYGB had too few patients to be meaningful. It is probably because the MBSAQIP database is not likely to have large numbers of OAGB, a procedure not endorsed by the American Society for Metabolic and Bariatric Surgery.

Global 30-day outcomes after bariatric surgEry duriNg thE COVID-19 pAndemic (GENEVA) study [9] is a large, multinational, observational study evaluating 30-day morbidity and mortality of bariatric and metabolic surgery (BMS) during the Coronavirus Disease-2019 (COVID-19) pandemic. The global reach of the study, a large number of patients, and significant numbers of OAGB procedures submitted to this study present a unique opportunity to compare 30-day morbidity and mortality of SG, OAGB, and RYGB in propensity score-matched cohorts.

## Methods

### Study design and population

The GENEVA study is an international, multicentre, observational cohort study of BMS performed between 1/05/2020 and 31/10/2020 [9]. The current study included all consecutive patients who underwent a primary SG or RYGB or OAGB during this period. Detailed methods have been published previously [9–11]. Data collection included patients’ demographics, details of surgery performed, and in-hospital as well as 30-day morbidity and mortality. Complications were categorised using the Clavien–Dindo (CD) Classification system for reporting surgical complications [12].

### Statistical methods

Only patients with a complete data entry were included in the analysis. Continuous data were presented as median and interquartile range. Frequencies were used to summarise categorical variables. To examine differences between the three individual procedure types, the Fisher’s exact test was used for categorical variables and Kruskal–Wallis analysis of variance testing for continuous variables.

Propensity score matching was completed in a step-wise fashion. Pairwise propensity matching was performed to robustly assess the quality of matching. Standardized mean difference (SMD) was used statistic to examine the balance of covariate distribution between treatment groups. Patients were matched using the following features: sex, Type 2 diabetes mellitus (T2DM) status (No diabetes; diet controlled; oral hypoglycaemics; insulin therapy), hypertension, hypercholesterolaemia, obstructive sleep apnoea, smoking status, age, and baseline body mass index (BMI).

The patients were matched against individuals that had other surgeries using the “nearest” method which utilises a greedy search to match each sample with their nearest neighbour. The distance was calculated using the Mahalanobis distance, which estimates the distribution closest for each point [13]. This procedure was performed in R (R Core Team 2021) using the Matchlt package [14, 15]. The outcome variable was the presence of a complication at 30-days follow-up.

Multivariate analysis was performed to strengthen the resulting statistics from univariate analysis, correcting the influence of each variable on the outcome measured. Multivariate models were created using all the variables used for propensity score matching plus ethnicity (white ethnicity vs other ethnic groups), presence of any co-morbidity, and other unspecified co-morbidity (other than those listed above). Patients were then analysed using a generalised linear model in R (R Core Team 2021) [14].

## Results

A total of 470 surgeons from 179 centres in 42 countries submitted data on 7092 adult patients who underwent primary BMS between 1st May 2020 and 31st October 2020 at the participating centres. Of these, complete 30-day morbidity and mortality data were available for 7084 (99.88%) by the 10th of December 2020.

### Basic demographics

Of the 7084 patients, 300 patients underwent other procedures and were excluded. A further 14 patients were excluded due to missing values. Complete data were available for a total of 6,770 patients who underwent a primary SG or RYGB or OAGB (SG *n* = 3983; RYGB *n* = 2085, OAGB *n* = 702). Demographic details for all the patients, who underwent any of these three primary procedures are included in Table 1. There were multiple significant differences in baseline demographics between the three groups as detailed in Table 1. RYGB patients were significantly older than patients in the other two cohorts while patients receiving OAGB were more likely to suffer from co-morbidities (Table 1). Patients undergoing SG had the lowest rate of each of these co-morbidities as detailed in Table 1.

**Table 1:** Baseline demographics of all patients undergoing a primary SG or RYGB or OAGB (unmatched cohort—14 patients excluded due to incomplete data).

Characteristic	SG (*n* = 3983)	OAGB (*n* = 702)	RYGB (*n* = 2085)	*p* value
Median Age (years)	38 (29.2–47.0)	40 (33.0–50.0)	43 (34–52)	<0.001^a^
Median BMI (kg/m^2^)	41.91 (38.14–46.77)	43.11 (38.87–48.77)	41.87 (38.67–45.73)	<0.001^a^
Sex (Female)	2883 (72%)	496 (71%)	1589 (76%)	<0.001^b^
Non-Smoker	2906 (73%)	528 (75%)	1502 (72%)	0.24^b^
T2DM	649 (16%)	229 (33%)	484 (23%)	<0.001^b^
Diet controlled	204 (5%)	74 (11%)	107 (5%)	
Oral hypoglycaemics	370 (9%)	106 (15%)	277 (13%)	
Insulin therapy	75 (2%)	49 (7%)	100 (5%)	
Hypercholesterolaemia	763 (19%)	224 (32%)	476 (23%)	<0.001^b^
Hypertension	1101 (28%)	272 (39%)	720 (35%)	<0.001^b^
Obstructive sleep apnoea	975 (24%)	229 (33%)	517 (25%)	<0.001^b^

*SG* sleeve gastrectomy, *RYGB* Roux-en-Y gastric bypass, *OAGB* one anastomosis gastric bypass, *BMI* body mass index, *T2DM* Type 2 diabetes mellitus.

^a^Kruskal–Wallis test.

^b^Fisher’s exact test.

### 30-day morbidity and mortality in the full cohort (unmatched; Table 2)

The overall complication rate was 6.7% (452/6770) (Table 2). RYGB patients had the highest rate of any complication during the 30-day follow-up (8.0% with RYGB vs 7.5% for OAGB and 5.8% for SG (*p* = 0.006)). There were seven post-operative mortalities (0.1%) (4 with SG, 3 with OAGB, and nil with RYGB; *p* = 0.016; Fisher’s exact test).

**Table 2:** Complications according to primary procedure and CD (Clavien–Dindo) classification system in the full unmatched cohort.

Characteristic	SG (*n* = 3983)	OAGB (*n* = 702)	RYGB (*n* = 2085)	*p v*alue
All 30-day complications	233 (5.8%)	53 (7.5%)	166 (8.0%)	0.006^a^
CD 0	3750 (94.1%)	649 (92.4%)	1919 (92.0%)	–
CD 1	84 (2.1%)	11 (1.6%)	62 (3.0%)	–
CD 2	63 (1.6%)	17 (2.4%)	48 (2.3%)	–
CD 3.1	16 (0.4%)	7 (1.0%)	8 (0.4%)	–
CD 3.2	50 (1.3%)	12 (1.7%)	31 (1.5%)	–
CD 4.1	13 (0.3%)	3 (0.4%)	15 (0.7%)	–
CD 4.2	3 (0.1%)	0	2 (0.1%)	–
CD 5 (Mortality)	4 (0.1%)	3 (0.4%)	0	0.016^a^

*CD* Clavien–Dindo, *SG* sleeve gastrectomy, *OAGB* one anastomosis gastric bypass, *RYGB* Roux-en-Y gastric bypass.

^a^Fisher’s exact test.

### Multivariate analysis of unmatched cohort (Table 3)

**Fig. 1:**
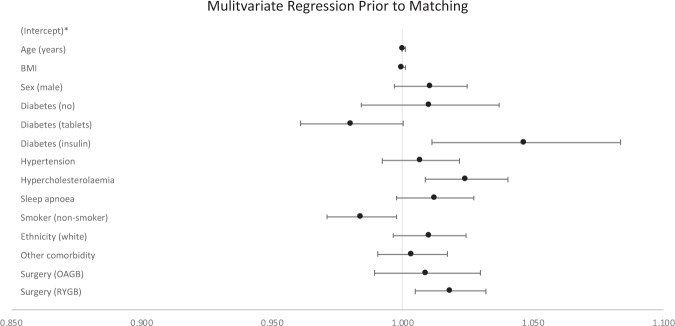
Multivariate regression results prior to patient matching.

On multivariate regression modelling, insulin-dependent T2DM (OR 1.047; 95% CI 1.011–1.083), and hypercholesterolaemia (OR 1.024; 95% CI 1.009–1.040) (Table 3 and Fig. 1) were associated with increased 30-day complications. Being a non-smoker was associated with reduced complication rates (OR 0.984; 95% CI 0.971–0.998). When compared to SG as the reference category, RYGB, but not OAGB, was associated with an increased rate of 30-day complications (OR 1.018; 95% CI 1.005–1.032 for RYGB and OR 1.009; 95% CI 0.989–1.030 for OAGB).

**Table 3:** Results of multivariate logistic regression on full unmatched cohort.

Variable	Unmatched patients	
	Odds ratio	95% CI
Age	1.000	1.000–1.001
BMI	1.000	0.999–1.001
Sex (Male)	1.011	0.997–1.025
Diabetes (No)	1.010	0.984–1.037
Diabetes (oral hypoglycaemics)	0.981	0.961–1.000
Diabetes (insulin)	1.047*	1.011–1.083
Hypertension	1.007	0.992–1.022
Hypercholesterolaemia	1.024*	1.009–1.040
Obstructive Sleep Apnoea	1.012	0.998–1.027
Non-Smoker	0.984*	0.971–0.998
White ethnicity	1.010	0.996–1.025
Other co-morbidity	1.004	0.991–1.017
Surgery (OAGB)	1.009	0.989–1.030
Surgery (RYGB)	1.018*	1.005–1.032

*BMI* body mass index, *OAGB* one anastomosis gastric bypass, *RYGB* Roux-en-Y gastric bypass, *CI* confidence interval.

*Significant values where *p* < 0.05.

### 30-day morbidity and mortality in the propensity score-matched cohort (Tables 4–6)

#### SG vs OAGB

In total, 702 pairs were matched with a reduction in SMDs for all matched variables (8/8) (Table 4). The overall complication rate in the SG group was 51 (7.3%) as compared to 53 (7.5%) in the OAGB group (Table 7). The difference was not significant (*p* = 0.68).

**Table 4:** A comparison of sleeve gastrectomy (SG) and one anastomosis gastric bypass (OAGB) before and after propensity score matching.

Characteristic	SG (702)	OAGB (702)	Standardised difference pre-matching (95% CI)	Standardised difference post-matching (95% CI)
Median Age (years)	40 (33–49)	40 (33–50)	0.203 (0.122–0.283)	0.011 (−0.093–0.116)
Median BMI (kg/m^2^)	42.87 (38.79–48.45)	43.11 (38.87–48.77)	0.132 (0.052–0.212)	0.017 (–0.087–0.122)
Sex (Female)	501 (71%)	496 (71%)	0.038 (−0.042–0.119)	0.016 (−0.089–0.12)
Non-Smoker	527 (75%)	528 (75%)	0.051 (−0.029–0.132)	0.003 (−0.101–0.108)
T2DM	229 (33%)	229 (33%)	0.407 (0.326–0.488)	0 (−0.105–0.105)
Diet controlled	74 (11%)	74 (11%)		
Oral hypoglycaemics	106 (15%)	106 (15%)		
Insulin therapy	49 (7%)	49 (7%)		
Hypercholesterolaemia	224 (32%)	224 (32%)	0.296 (0.215–0.376)	0 (−0.105–0.105)
Hypertension	270 (38%)	272 (39%)	0.237 (0.157–0.318)	0.006 (−0.099–0.11)
Obstructive sleep apnoea	230 (33%)	229 (33%)	0.181 (0.101–0.261)	0.003 (−0.102–0.108)

*SG* sleeve gastrectomy, *OAGB* one anastomsis gastric bypass, *T2DM* Type 2 diabetes mellitus, *CI* confidence interval.

**Table 5:** A comparison of sleeve gastrectomy (SG) and Roux-en-Y gastric bypass (RYGB) before and after propensity score matching.

Characteristic	SG (2085)	RYGB (2085)	Standardised difference pre-matching (95% CI)	Standardised difference post-matching (95% CI)
Median Age (years)	42.00 (33.00–51.00)	43.00 (34.00–52.00)	0.362 (0.309–0.415)	0.069 (0.008–0.129)
Median BMI (kg/m^2^)	42.19 (38.70–46.14)	41.87 (38.67–45.73)	0.096 (0.043–0.149)	0.064 (0.003–0.125)
Sex (Female)	1604 (77%)	1589 (76%)	0.088 (0.035–0.141)	0.017 (−0.044–0.078)
Non-smoker	1499 (72%)	1502 (72%)	0.021 (−0.032–0.074)	0.003 (−0.058–0.064)
T2DM			0.215 (0.161–0.268)	0.06 (−0.001–0.121)
Diet controlled	107 (5%)	107 (5%)		
Oral hypoglycaemics	277 (13%)	277 (13%)		
Insulin therapy	75 (4%)	100 (5%)		
Hypercholesterolaemia	477 (23%)	476 (23%)	0.09 (0.037–0.143)	0.001 (−0.06–0.062)
Hypertension	722 (35%)	720 (35%)	0.149 (0.096–0.202)	0.002 (−0.059–0.063)
Obstructive sleep apnoea	543 (26%)	517 (25%)	0.007 (−0.046–0.06)	0.029 (−0.032–0.089)

*CD* Clavien–Dindo, *SG* sleeve gastrectomy, *RYGB* Roux-en-Y gastric bypass, *CI* confidence interval, *BMI* body mass index, *T2DM* Type 2 diabetes mellitus.

**Table 6:** A comparison of one anastomosis gastric bypass (OAGB) and Roux-en-Y gastric bypass (RYGB) before and after propensity score matching.

	SG (*n* = 702)	OAGB (*n* = 702)	*p* value^a^	SG (2085)	RYGB (2085)	*p* value^a^
30-day complication	51 (7.3%)	53 (7.5%)	0.68	127 (6.1%)	166 (7.9%)	0.09
CD 0	651 (92.7%)	649 (92.4%)		1958 (94%)	1919 (92%)	
CD 1	18 (2.6%)	11 (1.6%)		41 (2%)	62 (3%)	
CD 2	10 (1.4%)	17 (2.4%)		36 (2%)	48 (2%)	
CD 3.1	7 (1.0%)	7 (1.0%)		10 (0%)	8 (0%)	
CD 3.2	10 (1.4%)	12 (1.7%)		25 (1%)	31 (1%)	
CD 4.1	3 (0.4%)	3 (0.4%)		9 (0%)	15 (1%)	
CD 4.2	1 (0.1%)	0		3 (0%)	2 (0%)	
CD 5	2 (0.3%)	3 (0.4%)		3 (0%)	0 (0%)	

*CD* Clavien–Dindo, *SG* sleeve gastrectomy, *OAGB* one anastomosis gastric bypass, *RYGB* Roux-en-Y gastric bypass.

^a^Fisher’s exact test.

**Table 7:** Complications in propensity score-matched populations.

	SG (702)	OAGB (702)	*p* value^a^	SG (2085)	RYGB (2085)	*p* value^a^	OAGB (702)	RYGB (702)	*p* value^a^
30-day complication	51 (7.3%)	53 (7.5%)	0.68	127 (6.1%)	166 (7.9%)	0.09	53 (7.5%)	53 (7.5%)	0.07
CD 0	651 (92.7%)	649 (92.4%)		1958 (94%)	1919 (92%)		649 (92%)	649 (92%)	
CD 1	18 (2.6%)	11 (1.6%)		41 (2%)	62 (3%)		11 (2%)	23 (3%)	
CD 2	10 (1.4%)	17 (2.4%)		36 (2%)	48 (2%)		17 (2%)	10 (1%)	
CD 3.1	7 (1.0%)	7 (1.0%)		10 (0%)	8 (0%)		7 (1%)	4 (1%)	
CD 3.2	10 (1.4%)	12 (1.7%)		25 (1%)	31 (1%)		12 (2%)	9 (1%)	
CD 4.1	3 (0.4%)	3 (0.4%)		9 (0%)	15 (1%)		3 (0%)	7 (1%)	
CD 4.2	1 (0.1%)	0		3 (0%)	2 (0%)		0 (0%)	0 (0%)	
CD 5	2 (0.3%)	3 (0.4%)		3 (0%)	0 (0%)		3 (0%)	0 (0%)	

*CD* Clavien–Dindo, *SG* sleeve gastrectomy, *OAGB* one anastomosis gastric bypass, *RYGB* Roux-en-Y gastric bypass.

^a^Fisher’s exact test.

#### SG vs RYGB

In total, 2085 pairs were matched with a reduction in SMDs for seven of the eight matched variables (Table 5). The overall complication rate in the SG group was 127 (6.1%) as compared to 166 (7.9%) in the RYGB group (Table 7). The difference was not significant (*p* = 0.09).

#### OAGB vs RYGB

In total, 702 pairs were matched with a reduction in SMDs in four of the eight matched variables (4/8) (Table 6). The overall complication rate in both the groups was the same 53 (7.5%; *p* = 0.07; Table 7).

## Discussion

This study shows that there is no significant difference in 30-day morbidity and mortality of SG, RYGB, and OAGB in propensity score-matched cohorts from a large, global dataset collected during the COVID-19 pandemic. Though RYGB was associated with higher 30-day morbidity in comparison with reference SG (OR 1.018; 95% CI 1.005–1.032) in the unmatched cohort on multivariate analysis, the difference disappeared after propensity score matching (*p* = 0.09). In comparison, OAGB was not associated with higher 30-day morbidity in comparison with SG on either multivariate analysis (OR 1.009; 95% CI 0.989–1.030) or propensity score-matched comparison (*p* = 0.68).

RCTs comparing different bariatric procedures often have weight loss [2] or diabetes control [16] as their endpoints. Some [16] do not even clearly report 30-day morbidity and mortality with different bariatric procedures let alone classifying surgical complications adequately according to the widely used and accepted CD Classification [12]. We cannot, therefore, derive any scientifically valid conclusions regarding complication rates of different procedures from these RCTs.

Outside of RCTs, perceptions regarding relative safety and efficacy of different procedures for different patient groups may introduce potential bias. For example, in this study, we found 33.0% of patients undergoing OAGB were suffering from T2DM compared to 23.0% undergoing RYGB and 16.0% undergoing SG in the unmatched cohorts. This selection bias may be partly accounted for by fact that the randomised studies have shown superior (non-significant as they were not powered to evaluate these) outcomes in terms of diabetes improvement with OAGB in comparison with RYGB [2] and with RYGB in comparison with SG [17]. This is important because T2DM is known to be associated with complications after bariatric surgery [18, 19] and in our study, Insulin-dependent T2DM was independently associated with 30-day morbidity on multivariate analysis of the unmatched cohort.

Similarly, in the unmatched cohort, hypercholesterolaemia was present in 19.0% of patients undergoing SG in comparison with 32.0% of those undergoing OAGB and 23.0% of those undergoing RYGB. However, after matching, in the analysis of SG and OAGB, the hypercholesterolaemia rates in the two groups were the same at 32.0 and 23.0% in the analysis of SG and RYGB. This is also important because hypercholesterolaemia was independently associated with 30-day morbidity on multivariate analysis of the unmatched data and differences in hypercholesterolaemia rates in the unmatched cohort may well have accounted for some of the observed differences in 30-day morbidity. Others [20] have also found dyslipidaemia to be a predictor of complication after bariatric surgery.

Standardised mean differences in age, BMI, sex, smoking status, hypertension rates all reduced after matching for both the matched comparisons involving SG in this study. Given that all of these characteristics are known to be associated with increased morbidity after bariatric surgery [21–29], differences in these baseline characteristics may have been in part responsible for why the observed difference in morbidity between SG and RYGB or OAGB disappeared after matching. At the same time, and probably because of the fewer number of bypass procedures in the GENEVA database, matching failed to reduce SMDs for age, sex, smoking status, and hypertension in the comparison between OAGB and RYGB in this study. This may partly account for the observed lack of difference in 30-day morbidity between the two procedures. Future studies on this topic need to be mindful of this.

There is no published study comparing the 30-day morbidity of SG with that of OAGB in propensity score-matched cohorts. This may be due to continued reservations [30] amongst some surgeons about OAGB. Furthermore, and as mentioned above in the Introduction section, there is only one propensity score-matched study [8] in the scientific literature comparing the 30-day safety of OAGB with that of any other procedure (RYGB in this case) and that study only had 279 pairs of OAGB and RYGB. One could argue this is not a large enough sample to study differences in morbidity.

There is only one study [7] in the published literature comparing the 30-day morbidity of SG with that of RYGB. Interestingly that study showed lower complication rates with SG in contrast to our findings. It is however worth noting that these authors do not report standardised mean difference in propensity score-matched populations and given the large numbers matched, it was inevitable that their matching was not perfect with significant difference between the matched populations with regards to important confounding variables such as age, BMI, smoking, insulin-dependent T2DM, etc.

This study represents the first large propensity-matched comparison of 30-day morbidity and mortality of SG, RYGB, and OAGB. This data was collected from a large worldwide collaborative study of real-world bariatric surgical practice. Data completion rates were extremely high with 30-day follow data available for 97.9% of patients across the entire cohort and this represents a significant strength of this study.

Non-randomised design and self-reported complication rates are two major weaknesses of this study. However, it is not easy to randomise to different procedures with 30-day morbidity as an endpoint and anonymous data collection strategy used in this study may have diminished the desire to under-report complications. Another weakness of this study is that differences in complication rates, though statistically not significant, maybe clinically relevant. Indeed, larger studies may even find statistical significance.

## Conclusion

The present analysis shows that there is no significant difference in 30-day morbidity and mortality of SG, OAGB, and RYGB in propensity-matched cohorts.

## Supplementary information

Appendix
